# Polyglutamine Disease Modeling: Epitope Based Screen for Homologous Recombination using CRISPR/Cas9 System

**DOI:** 10.1371/currents.hd.0242d2e7ad72225efa72f6964589369a

**Published:** 2014-04-15

**Authors:** Mahru C. An, Robert N. O'Brien, Ningzhe Zhang, Biranchi N. Patra, Michael De La Cruz, Animesh Ray, Lisa M. Ellerby

**Affiliations:** The Buck Institute for Research on Aging, Novato, California, USA; The Buck Institute for Research on Aging, Novato, California, USA; The Buck Institute for Research on Aging, Novato, California, USA; School of Applied Life Sciences, Keck Graduate Institute, Claremont, California, USA; School of Applied Life Sciences, Keck Graduate Institute, Claremont, California, USA; School of Applied Life Sciences, Keck Graduate Institute, Claremont, California, USA; The Buck Institute for Research on Aging, Novato, California, USA

## Abstract

We have previously reported the genetic correction of Huntington’s disease (HD) patient-derived induced pluripotent stem cells using traditional homologous recombination (HR) approaches. To extend this work, we have adopted a CRISPR-based genome editing approach to improve the efficiency of recombination in order to generate allelic isogenic HD models in human cells. Incorporation of a rapid antibody-based screening approach to measure recombination provides a powerful method to determine relative efficiency of genome editing for modeling polyglutamine diseases or understanding factors that modulate CRISPR/Cas9 HR.

## Introduction

The ability to model disease in cells and animals through targeted modification of the genome represents a powerful approach to understanding genetic and molecular mechanisms underlying disease states 1. New advances in genome editing technology promise to vastly improve the set of tools with which to develop engineered lines 2.

The type II prokaryotic CRISPR (clustered regularly interspaced short palindromic repeats)-associated 9 (Cas9) nucleases are uniquely targeted to a specific genetic locus by a single-guide RNA (gRNA). Specificity of the gRNA is established through a 20 nucleotide homology to the target region which is followed by a 5’-NGG protospacer adjacent motif (PAM), and the complexed Cas9 cleaves the DNA upstream of the PAM 3. The ability to designate sequence specificity to the genomic target based on the customizable base pairing affinity of user-designed gRNAs represents a cost effective, versatile system with which to introduce double stranded breaks in the host genome. Applying this tool, as recent studies have shown, to enhance the frequency of donor DNA-mediated homologous recombination (HR) events may significantly improve the extent to which these types of approaches can be utilized to tailor engineer genomic loci in both cells and organisms 4 ^-^ 7. Specificity of the Cas9 can be further refined by using the mutant Cas9 D10A which results in partial inactivation of the nuclease catalytic activity. This mutation converts the wild-type enzyme which produces double-stranded DNA breaks into a "nickase" enzyme that produces single-stranded breaks at the target site. The Cas9 D10A mutation lowers the rate of nonhomologous end joining (NHEJ) and favors DNA repair by HR at the targeted site.

One way in which this type of targeted genome editing can be used is the generation of mutations in an isogenic background to model disease in human cell lines such as patient derived induced pluripotent stem cells (iPSCs). The effective study of disease states *in vitro* can be greatly enhanced by the ability to easily introduce defined modifications to the genome of human cells, particularly when combined with the added ability to readily differentiate these cells into disease-relevant subtypes 8. Our previous work has established the utility of gene targeting via traditional homologous recombination (HR) to genetically correct the expanded disease causing polyglutamine (polyQ) mutation within exon 1 of the huntingtin (*HTT*) gene in HD patient-derived iPSCs 9. Targeted HR-mediated genetic correction of expanded polyQ region to normal length resulted in the reversal of HD-associated phenotypes in the corrected cell lines, providing a useful isogenic cell model system for the study of expanded HTT in human cells 9. While our work demonstrated the feasibility of generating gene modified cell lines through traditional HR based methods, the use of Cas9 nuclease based tools greatly enhances the ability to develop multiple allelic mutations in an efficient manner. We have now adopted Cas9 nuclease based tools to enhance frequency of HR events at this previously characterized locus, while establishing a novel antibody-based approach to measure the relative rate of gene targeting events within our system. The antibody screen using the epitope for the polyQ expansion allows the rapid generation of an allelic series harboring various repeat lengths. The use of the Cas9 D10A lowers the rate of NHEJ and favors HR at the targeted site.

## Results

In order to develop technology to rapidly make allelic CAG expanded isogenic lines of polyQ disease iPSCs, we have designed a gain-of-epitope screen to assess targeted recombination events for construction of these lines. We used a modified version of our previous targeting vector 3 that incorporates a disease containing CAG/CAA sequence of 97 repeats and a neomycin resistance cassette (**Fig. 1a**) to genetically modify the polyQ repeat length via homologous recombination. To target Cas9 nuclease activity to the region of *HTT* exon 1, we have generated two gRNA sequences that are unique in the genome and which cut within 100 bp of the CAG region of exon 1 of the *HTT* gene. The specificity of these *HTT* gRNAs was assessed, and both were confirmed to be unique targets in the genome 10 (**Fig. 1a, Supplementary Fig. 1a**). We assessed the ability of these *HTT* gRNA constructs to enhance targeting donor mediated recombination in 293F cells. Cells were transfected with either *HTT* gRNA1, *HTT* gRNA2, AAVS1-1 gRNA (control targeting different site), or empty vector, and co-transfected with or without targeting donor construct and a human codon-optimized Cas9 expression plasmid, hCas9 11. Transfection and processing steps were done using a protocol that would allow for both western blot analysis and colony number counting over multiple conditions and several replicates (**Fig. 1b**). Experimental replicates were established at time of transfection, and the cells for colony counting and western blot analysis are representative of the same original transfection replicate maintained in selective antibiotic G418.

**Homologous recombination strategy for introduction of the polyQ expansion in HTT exon 1 into wild type cells.**
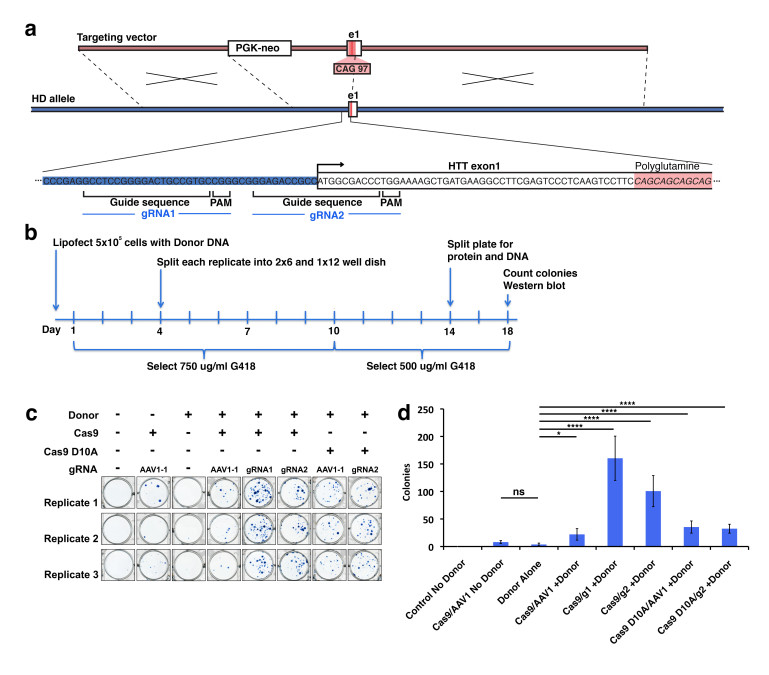
(a) Homologous donor containing a neomycin resistance cassette 1.5 kb upstream of exon 1 as described previously was used as a donor. Cas9 guide RNA (gRNA) sites were designed near the translational start site of the gene. *HTT* gRNA sequences were designed based upon criteria described previously. (b) Experimental design for treatment of 293F cells with Cas9 constructs. Cells were lipofected at day 0, placed on selection at day 1 and remained on selection until the cells were harvested at day 18. (c) At day 18 cells were fixed and stained with methylene blue/methanol and scanned on a flatbed scanner. Resulting scans were analyzed with the colony counting function in ImageJ. (d) There was a significant increase in colonies in all cells treated with Cas9, *HTT* gRNA and donor. This increase was most pronounced in cells expressing WT Cas9 and *HTT* specific gRNAs. The increase in colony number was donor-dependent.

We first counted neomycin resistant 293F colonies to determine relative donor construct integration between transfection replicates. We fixed and stained colonies using methylene blue (**Fig. 1c**). Quantification of methylene blue stained clonal colonies revealed striking differences in the number of neomycin resistant colonies (**Fig. 1d**). Cells transfected with *HTT* gRNA1 or 2 in the presence of donor and Cas9 expression show a statistically significant increase in colony number over Cas9 background or donor alone. This suggests that the donor integration is significantly enhanced in a Cas9/gRNA-mediated manner. As expected mock transfected cells show no surviving colonies, Cas9 + control gRNA transfected cells show a low level of background neomycin resistance which is higher than donor alone, but is not statistically significant. We also used nickase Cas9 D10A, a form of the enzyme where one of two nuclease domains is inactivated, creating a single stranded nick as opposed to a double stranded break. Cells transfected with the mutated Cas9 D10A in the presence of donor and *HTT* gRNA2 also show a significant increase in colony number over donor alone (**Fig. 1c,d**). This increase was slightly lower than the WT Cas9 transfections, and not significantly higher then the control AAVS1-1 gRNA but upon further screening of western and Southern blot analysis proved substantial-see below****. This underscores the importance of having an expedient additional screening method to detect targeted insertions, as the neomycin only screen would result in the Cas9 D10A appearing ineffective.

While colony number is a strong indicator of construct integration, many factors including random integration events can result in colony resistance to selective antibiotics. Therefore, we developed a rapid epitope based screen for recombination. The monoclonal antibody 1C2 detects the homopolymeric glutamine stretch epitope MAB1574 originally from TATA box binding protein (TBP) containing a 38 glutamine stretch, and has been shown to have strong specificity to the expanded polyQ length forms of HTT and other polyQ expansion disease proteins^12^. 293F cells carry wild type non-expanded HTT and therefore targeted integration of expanded HTT is reflected by a gain of 1C2-recognizable epitope that can easily be measured by western blot analysis of clonal or mixed clonal cell populations. We took advantage of this characteristic to allow us to measure relative levels of targeted expansion in our 293F cells. Protein lysates were collected and equal protein amounts were analyzed by western blot for relative immunoreactivity to 1C2 (**Fig. 2**). Remarkably, Cas9/*HTT* gRNA/donor transfected cell lysates show distinct, measurable levels of 1C2 immunoreactivity, with a significant increase in 1C2 levels relative to control gRNA (**Fig. 2a,b**). Similarly, nickase Cas9 D10A/*HTT* gRNA/donor transfected cell lysates showed comparably high levels of 1C2 immunoreactivity relative to control (Fig. 2a,c). A separate experiment using the same system with no selection in 293T cells yielded appreciable 1C2 signal in all Cas9 and *HTT* gRNA treated conditions (**Supplementary Fig. 2**). Additionally, we detected comparable 1C2 immunoreactivity in lysates from 293F cells co-transfected with donor and two independent *HTT* TALEN pairs that had also been characterized for this locus (**Fig. 2a, Supplementary Fig. 1b,c**). 1C2 levels are reflective of abundance within the total colony pool of a sample. While differences in clonal numbers make it difficult to compare absolute frequencies between WT Cas9, nickase Cas9 D10A, and TALEN, these 1C2 levels clearly reflect a marked increase in polyQ expanded cells compared to the control, offering a convenient way to verify target site homologous recombination events. To gauge the representation of 1C2 positives in the neomycin resistant pool, we performed clonal 1C2 western analysis on individual colonies picked from Cas9 D10A/*HTT* gRNA1/donor transfected cell populations after neomycin selection. All 12 clones picked showed a strong 1C2 band compared to control gRNA and untransfected controls (**Fig. 2d**). Taken together, we conclude that CRISPR-assisted HR results in an increase in colonies over TALENs, with a similar rate of homologous recombination of those colonies.

**Pooled 293F neomycin-resistant cells were analyzed by western blot.**
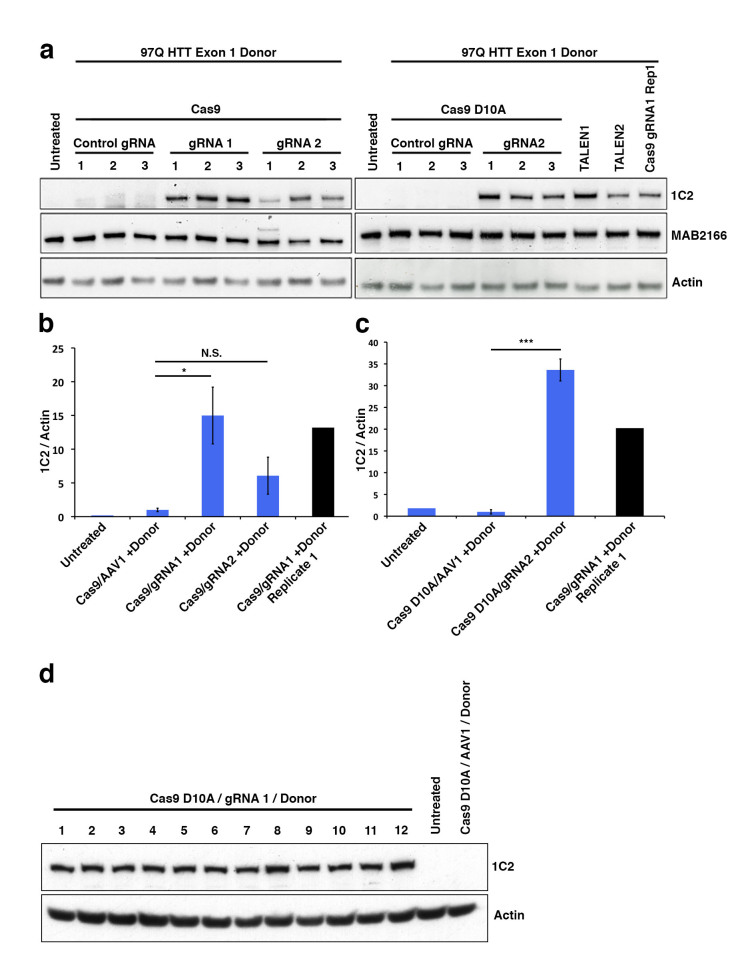
(a) To identify 1C2-reactive full-length HTT protein indicative of homologous recombination a western blot assay was utilized. Total HTT (MAB2166) and β-actin are also shown. (b) Quantitated levels of 1C2 signal normalized to β-actin are significantly increased in cells treated with WT Cas9 and HTT specific gRNAs realtive to cells treated with a gRNA targeting a different (AAVS1) locus in the genome. (c) Cas9 D10A show significant increase in *HTT* gRNA2-mediated recombination relative to control, AAVS1 gRNA assisted cells. (d) 1C2 western blot analysis of individual clones (1-12) transfected with Cas9 D10A, *HTT* gRNA1, and donor compared with control gRNA and untransfected controls.

To further support these findings, we transfected HD patient derived HD-iPSC cells with the 97Q targeting donor construct in the presence or absence of WT Cas9 and *HTT* gRNA2 as described previously 9. Cells were selected for 3 weeks on G418 in feeder free medium, before individual clonal colonies were manually picked and transferred to individual wells for expansion and genomic DNA analysis (**Fig. 3a**). The HD-iPSC line carries HTT polyQ lengths of 19 and 72 repeats which can be measured by PCR amplification. Using separate primer sets to amplify either the endogenous exon1 CAG repeat region or the modified exon1 97Q CAG repeat region, we determined that 2 of 36 neomycin resistant colonies picked in the donor only transfected cells showed a loss of endogenous CAG product coupled with a gain of the modified 97Q band while from cells transfected with Cas9, *HTT* gRNA2, and donor, we found 8 of 60 such colonies (**Fig. 3b,d**). Western blot analysis showed 7 of these colonies were immunoreactive to 1C2 antibody (data not shown). Further genomic analysis of the clonal DNA by Southern blot analysis showed that neither of the two donor only candidates are positive for targeted recombination, while 7 out of 8 of the Cas9/*HTT* gRNA2/donor transfected colonies are positive for this specific recombination event consistent with the 1C2 screen (**Fig. 3c,d**). The observed recombination rates (approx. 12%) with Cas9 and *HTT* gRNA are remarkably higher in comparison to the previous frequencies that we have reported at this locus by traditional homologous recombination (1.0%).

**Cas9-mediated homologous recombination in iPSCs.**
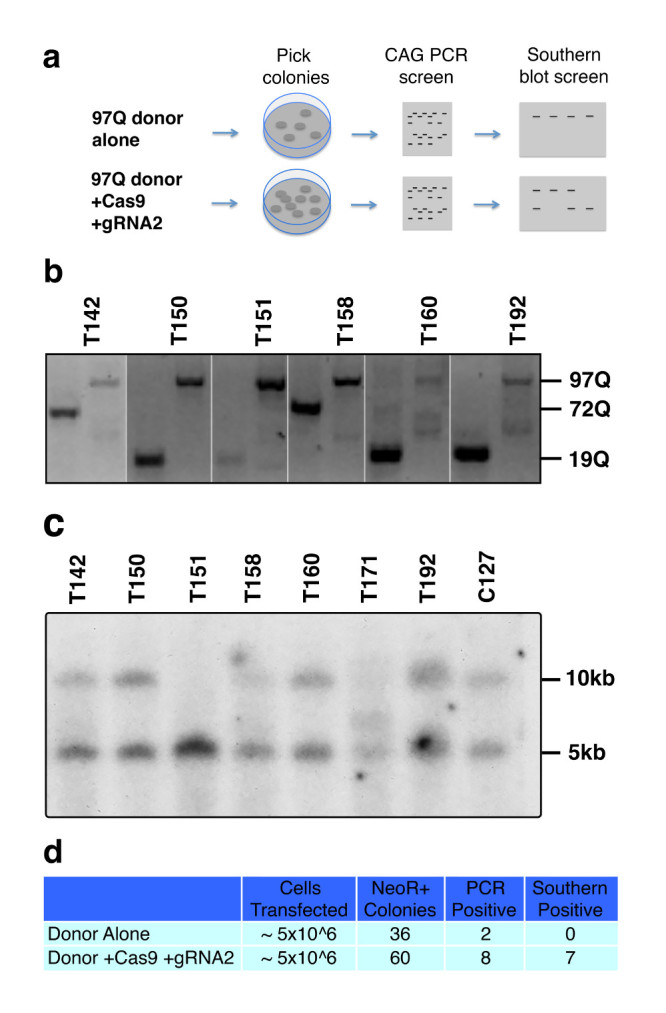
(a) Experimental design and screening process for transfected iPSCs. (b) Candidates for potential targeted integration of *HTT* exon 1 97Q donor knockin events are screened first by PCR. Separate primer sets specifically amplify either the endogenous CAG length (first lane of each clone) or the introduction of the modified 97Q exon 1 (second lane). Candidates were identified by the loss of one endogenous band coupled with gain of 97Q band. (c) Genomic DNA from iPSC clones are digested with HindIII and probed with a 300 bp radiolabeled probe that is specific to a region external to the targeting donor sequence. Successful integration of targeting donor sequence results in the introduction of a HindIII site that results in truncation of the non-targeted fragment length from 10 kb to 5 kb. (d) Table summarizing frequency of HR and conditions.

## Discussion

We show that CRISPR/Cas9 system can be utilized to perform homologous recombination in human cells to generate a HD isogenic allelic series (21, 72, 97 CAG). In our study, we compared the Cas9 vs. Cas9 D10A enzymes for their ability to mediate this event using two distinct endpoints. The use of Cas9 D10A is likely to be more selective with less off target effects. When we compared CRISPR-assisted HR using methylene blue stained clonal colonies it appeared that the wildtype Cas9 was much more effective at mediating HR. However, when we analyzed the same experiment in our 1C2 western blot assay which only detects HR events at the Htt locus due to the introduction of the 97 CAG repeats, we found that both Cas9 and Cas9 D10A were similar in generating the 97CAG expansion at this site. It is likely that the wild-type Cas9 introduces off target invents that allow for more colonies resistant to neomycin than the Cas9 D10A enzyme.

Our results suggest that CRISPR-assisted homologous recombination is a useful technology that can be effective in enhancing donor-mediated gene targeting events in both 293 cells and human iPS cells. Utilizing an antibody-based method to screen for recombination events that alter structural epitopes or introduce new epitopes such as gain of 1C2 reactivity represents a novel method for identifying targeted candidates or assessing targeting efficiency in a polyclonal pool of cells without the use of an artificial reporter system. Indeed it may eliminate the need for single colony screening or extensive Southern blotting. Combining these two technologies, we are able to demonstrate the efficient generation of genetically modified human cell lines for study of HD. Given the growing excitement in the field of genome editing, our screen offers a novel antibody based method for screening for HR events and optimization of this technology 13 ^,^ 14. Using drug libraries or siRNA approaches with this screen could reveal novel pathways/modulators of HR. In summary, we provide further tools for understanding HR using the CRISPR/Cas9 system and for the creation of relevant polyQ disease models.

## Methods

**Generation of HTT exon 1 gRNA sequences. **Guide RNA sequences for the CRISPR nuclease system were designed as described in Mali et al.^11^. Briefly, sites that contained the sequence G(N_20_)GG near exon 1 of HTT were identified. Candidate sequences were then analyzed in BLAST to determine whether they were unique in the genome. Two *HTT* gRNA targets, uniquely present in the genome were identified, these sequences were cloned into the Church lab gRNA_Cloning Vector (addgene plasmid 41824) utilizing the primer annealing strategy published by the Church lab. Primers of sequences: TTTCTTGGCTTTATATATCTTGTGGAAAGGACGAAACACCG**CCTCCGGGGACTGCCGTGC **(gRNA 1 Forward) and GACTAGCCTTATTTTAACTTGCTATTTCTAGCTCTAAAAC**GCACGGCAGTCCCCGGAGGC** (gRNA 1 Reverse) and TTTCTTGGCTTTATATATCTTGTGGAAAGGACGAAACACCG**GAGACCGCCATGGCGACCC** (gRNA 2 Forward) and GACTAGCCTTATTTTAACTTGCTATTTCTAGCTCTAAAAC**GGGTCGCCATGGCGGTCTCC** (gRNA2 Reverse) were annealed and ligated into AflII-linearized gRNA_cloning vector by Gibson assembly. Resulting colonies were sequenced and maxiprepped with the Qiagen Plasmid Plus kit.

**Generation of TALENs.** TALENs 1 and 2 were designed to cleave within a 13 bp segment of the genomic DNA in the 5’ UTR region of *HTT*. The binding sites of both TALENs flank the predicted cleavage site. These two TALENs were selected from a pool of 10 TALENs designed for the same target site on the basis of efficient cleavage on a surrogate reporter plasmid in an *in vivo* transient transfection assay.

**Generation of HTT 97Q donor.** A modified 240 kb BAC (RP11-866L6) containing the 170 kb human HTT locus with an exon 1 containing 97 mixed CAG-CAA repeats 15 was modified stepwise using a Red/ET-based recombineering kit (Genebridges). First, a PGK-neo expression cassette flanked by FRT recognition sites was inserted 1.5 kb upstream of exon 1. The 20 kb fragment, including exon 1, inserted expression cassette, a 4.5 kb upstream short arm, and a 10 kb downstream long arm, were inserted into a modified pPNT vector (neo cassette removed) adjacent to the HSV-TK.

**Transfection and selection of 293F cells.** Low passage 293F cells (passage 8) were grown in the DMEM high glucose +NEAA +NaPyruvate +10% FBS and plated overnight on 6 well plates (Corning) at a density of 500,000 cells per plate in 2 ml media. Cells were then transfected with Lipofectamine 2000 (Life Technologies) according to manufacturer’s instructions. Briefly, 3 μg of DNA (1 μg Donor, 1 μg gRNA, 1 μg Cas9 or pCDNA3.1 empty vector) was diluted in 250 μL DMEM and mixed with 5 μL LF2000 diluted in 250 μL DMEM and allowed to incubate for 5 min prior to mixing. DNA-LF2000 complexes were then allowed to form for 30 min, and the mixture added to plates. After 24 h, media was replaced with media containing 750 μg/ml G418 (Life Technologies). Cells were passaged at day 4 and media replaced at days 7, 10 and 14. Cells were harvested or stained with methylene blue at day 18.

**Methylene blue staining and colony counting.** 6 well plates were collected (18 d) and stained using 0.2% (w/v) methylene blue in 50% methanol for 20 min. Plates were then washed extensively in milliQ H_2_0, allowed to dry and scanned on a flat-bed scanner. Images were then analyzed in ImageJ as followings: Color images were converted to binary. Particle size was set from 10-infinity. An identical circular ROI was used for all wells in order to exclude the side walls and other artifacts. Particles were measured and both counts and area fraction were entered into excel. Count data were analyzed by one-way ANOVA after transformation by taking the square root of the observed colony number, as is recommended for counts of colonies.

**Western analysis of neomycin resistant 293F cells.** Cells were harvested by scraping, spun down for 2 min at 1000xg and lysed in MPER (Thermo Scientific) containing protease inhibitors. 25 μg of each sample was loaded on a 12 well 4-12% Bis-Tris gel in MES buffer (Life Technologies) and run at 200 volts for 60 min. Protein was then transferred to a nitrocellulose membrane at 20 volts for 14 h. Membranes were blocked and probed with 1C2 (Millipore 1:1000) in 5% milk in TBST. After detection, blots were reprobed with antibodies against HTT (MAB2166, Millipore 1:1000) and β-actin (Cell Signaling 1:1000).

**Transfection 293T cells.** Low passage 293T cells (passage 8) were grown in the DMEM high glucose +NEAA +NaPyruvate +10% FBS and plated overnight on 6 well plates (Corning) at a density of 500,000 cells per plate in 2 ml media. Cells were then transfected with Lipofectamine 2000 (Life Technologies) according to manufacturer’s instructions. Briefly, 3 μg of DNA (1 μg Donor, 1 μg gRNA, 1 μg Cas9 or pCDNA3.1 empty vector) was diluted in 250 μL DMEM and mixed with 5 μL LF2000 diluted in 250 μl DMEM and allowed to incubate for 5 min prior to mixing. DNA-LF2000 complexes were then allowed to form for 30 min, and the mixture added to plates. Cells were grown for 6 days and harvested for western blot.

**Western analysis of unselected 293T cells.** Cells were harvested by scraping, spun down for 2 min at 1000 x g and lysed in MPER containing protease inhibitors. 25 μg of each sample was loaded on a 12 well 4-12% Bis-Tris gel in MES buffer (Life technologies) and run at 200 volts for 60 min. Protein was transferred to a nitrocellulose membrane at 20 volts for 14 h. Membranes were blocked and probed with 1C2 (Millipore 1:1000) in 5% Milk in TBST. After detection, blots were reprobed with antibodies against HTT (MAB2166, Millipore 1:1000) and β-actin (Cell Signaling 1:1000).

**Electroporation of hiPSCs.** Feeder-free HD-iPSCs were grown on matrigel (BD Biosciences) coated plates in mTeSR medium (Stem Cell Technologies). Cells were grown to 70-80% confluency and were pre-treated with 10 μM Y-27632 ROCK inhibitor for 1 h. Cells were then dissociated by TrypLE (Life Technologies) treatment for 2-3 min and spun down at 200 x g. Cells (1 x 10^6^) were resuspended in 100 μl nucleofector human stem cell solution 1 including supplement (Lonza) and immediately nucleofected with program A-027. Cells were transferred to 2 ml pre-warmed RPMI+20% KSR and incubated at 37^o^C for 15 min, then transferred to pre-warmed mTeSR on Matrigel coated plates at a density of 1-2 x 10^6^ cells per 10 cm plate. G418 at 50 μg/ml was added starting at 24 h post-nucleofection and continued for 21 d with daily changes of medium. Surviving colonies at 21 d were manually passaged by incubation with 0.4 mg/ml collagenase for 30-90 min, dissociation with a 200 μl pipette tip, and transfer to 96-well plates.

**PCR screen and Southern blot analysis. **Duplicates of iPSC clones were grown and passaged to 24-well plates. Cells were incubated overnight in lysis buffer, followed by precipitation in equal volume of isopropanol and 70% EtOH wash. DNA was resuspended in TE. The crude genomic preparation was screened first by PCR protocols amplifying the CAG repeat region of the *HTT *gene using separate sets of primers to amplify either the endogenous exon 1 CAG repeat sequence or the modified 97Q exon 1 CAG repeat sequence. Clones showing both the loss of an endogenous allele and the gain of expanded 97Q allele were tested by Southern blot analysis as described previously 9.

## Figures and Tables

**Supplemental:**
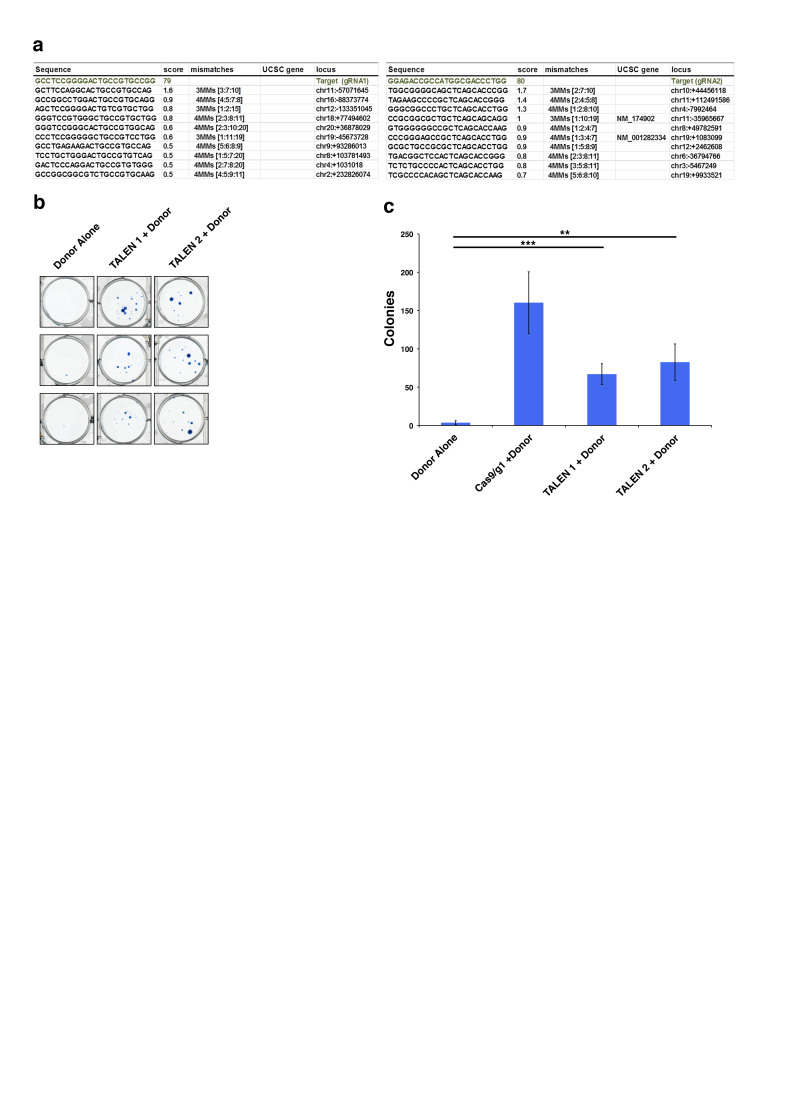
(a) Specificity and mismatch scores for *HTT* gRNA1 and 2 assessed by the CRISPR resources design tool^10^. (b) Methylene blue stained colonies of *HTT* TALEN pair 1 and 2 + donor transfected 293F cells compared to donor alone. (c) Quantification of colony number from *HTT* TALEN + donor transfected cells compared to donor alone and Cas9/*HTT* gRNA1 + donor transfected cells.

**Supplemental:**
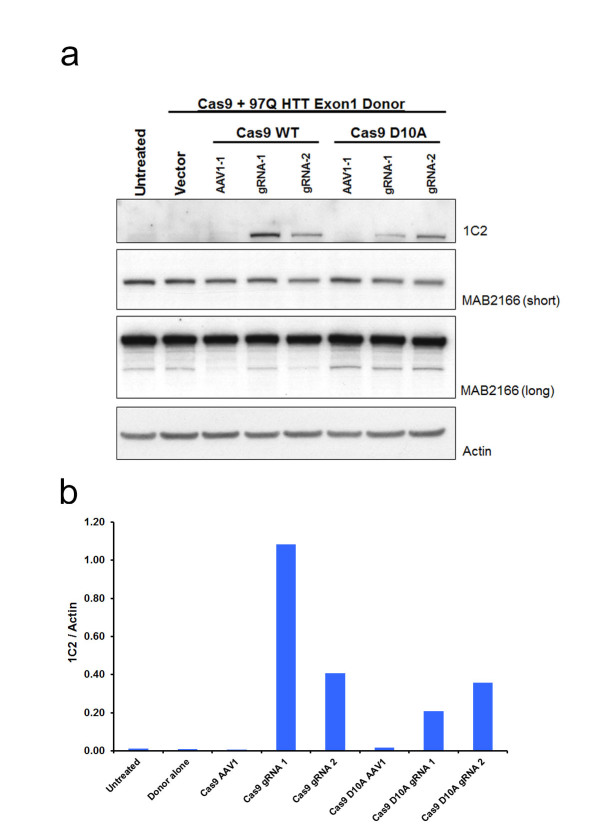
Pooled 293T resistant cells were analyzed by western blot 6 days after transfection with indicated conditions to identify 1C2-reactive full-length HTT protein indicative of homologous recombination. (a) Western blot confirms 1C2 signal is specific to Cas9 and *HTT* gRNAs, with little to no signal apparent in untreated, donor alone or donor/Cas9/AAV controls (b) Quantification of western data.
